# Protargol in Veterinary Surgery

**Published:** 1902-05

**Authors:** W. E. A. Wyman

**Affiliations:** Portland, Mich.


// THE JOURNAL OF COMPARATIVE MEDICINE AND VETERINARY ARCHIVES.

Vol. XXIII. *MAY, 1902.* No. 5.

### THE JOURNAL

### OF

### COMPARATIVE MEDICINE AND VETERINARY ARCHIVES.

### PROTARGOL IN VETERINARY SURGERY.

By W. E. A. Wyman, V.S.,
PORTLAND, MICH.

It is almost one year since the liberality of the Farbenfabriken of Elberfield Co., 40 Stone Street, New York city, began to place a practically unlimited supply of protargol at the disposition of the writer. The method of employment was either the pure powder, watery solution, bougie with cocoa butter, and finally ointment with lanolin. Dr. Eichengruen, the discoverer of protargol, in 1897 said: "We possess in it a preparation which contains silver, not only in a masked form, but also in organic combination—that is, instead of a salt or double salt, in firm combination with a proteid. This product, which is readily soluble in water, is not precipitated by alkaline sulphates, albumin, sodium chloride, or acids, and has a completely neutral reaction."

No other silver salt has such properties; for these reasons it ought to act deeply without irritating materially the tissues, and containing 8.3 of silver its extraordinary bactericidal properties are explained. Not being precipitated by the saline and albuminous constituents of wound secretions, its bactericidal action is not impaired, but active at all times.

Protargol is plainly a desiccant, holds granulations in check, and produces rapidly a firm scar, being distinctly superior to iodoform, lead, aluminum acetate, tannoform, aristol as a cicatrizant. It is of great value in infected wounds, especially those with a purulent infiltration of the surrounding tissues; in these its deeply penetrating qualities are beautifully demonstrated.

In the treatment of tubular ulcers or fistulæ it is almost a specific. In catarrhal and ulcerative affections of the ear it is easily the promptest agent of to-day, while its extraordinary cicatrizing action is demonstrated by the application of a 10 per cent. lanolin ointment to that often troublesome and frequent eczematous dermatitis of the heels vulgarly termed scratches.

Internally the writer has employed protargol twice in gastro-intestinal affections, and, as the perusal of the subsequent reports will show, with excellent results.

The following cases all refer to the horse unless otherwise stated:

1. *Glossitis*, cause unknown. Intraparenchymatous injections with 5 per cent. protargol solution, given once daily two days in succession; one abscess was opened on the fourth day. Complete recovery on the seventh day.

2. *Osteomyelitis*, region of interdental space lower maxilla, with perforation at inferior border, necrotic bone smell, caused by pressure of curb-bit. Curettement of fistulous tract, with daily injections of 20 per cent. protargol solution. Complete recovery on the tenth day. Fourteen such cases have been treated with protargol injections. Pain decreased invariably on the third to fourth day following the first injection.

3. *Empyema of frontal and maxillary sinus*, due to alveolar periostitis of fourth upper molar. All cases of several months' standing. Extraction of tooth and trephining of frontal and maxillary sinus. Irrigations of sinuses three times daily with 5 per cent. protargol solution. Discharge stopped on an average, after irrigating the sinuses, sixteen days.

Four such cases were treated with protargol. One of these had been treated by a number of gentlemen for about two years, but continued to exhibit a nasal discharge. In this case frontal and nasal sinuses were trephined, and since no neoformations of any sort became manifest after trephining the cavities, 10 per cent. protargol irrigations were ordered three times daily. At the end of one week the discharge had greatly diminished in quantity, and its purulent nature had been changed into a mucoid type. The second week 20 per cent. irrigations twice daily were ordered; at the end of the third week the animal was discharged as cured. Three months later the owner reported him as free from nasal discharge.

4. *Otitis externa.* The chronic form, commonly and improperly called canker of the ear, has always been a more or less troublesome ailment to the canine practitioner, especially those with profuse suppuration and great pain. The results obtained with protargol are simply marvellous. First cleanse the ear-duct with a warm alkaline solution, the writer giving bicarbonate of soda the preference. This is followed with an injection of alcohol, and after the parts are dry a 20 per cent. protargol solution is slowly injected into the ear duct three times daily. Some very sensitive animals will at this moment shake the head violently and possibly cry out, but the itching and pain subside almost at once. All cases treated so far, even those with excoriations and excessive granulations, made a prompt recovery under the protargol injections. The time consumed was from three days to one month. Among the forty-two cases of external otitis which recovered was one water spaniel that suffered with it for two years, and was shown the writer as a curiosity on account of the unbearable smell from his ears. The ear-ducts of this creature were filled with granulations and numerous ulcerations; after thorough curettement protargol did the rest.

5. *Poll-evil,* 5 cases. The third one only is worthy of special description. It had been operated at various places for the past three years; occasionally healed up, to break out anew in a few weeks. Examination showed necrosis of the ligamentum nuchæ, necrosis of the superior spinous process of the axis, also intermuscular phlegmon along the superior third of the neck. The treatment consisted of free and deep incisions along the fistulous tracts, cutting of the funicular portion of the ligamentum nuchæ, scraping away of all necrotic bone, insertion of setons wherever necessary to insure perfect drainage, and thrice daily irrigations with ½ per cent. formalin solution, followed by an injection of protargol 10 per cent., aquæ 40 per cent., glycerini 50 per cent. The animal was returned in three weeks and the setons removed. Seven months later the animal was seen again, the wounds having closed entirely six weeks from the day of operation. The time consumed in the protargol treatment of the other cases varied from fourteen days to five weeks.

6. *Fistula over the sternum.* The animal was bought with the fistula. Owner objected to radical operation, and was given the same protargol solution as subject 5, and was ordered to inject it three times daily. Fourteen days later he came for another supply, and finally reported the fistulous tract closed at the end of the fourth week.

7. *Fistulous withers.* Of these, eleven have been treated. Observations made show the absolute necessity of perfect drainage and removal of necrosed bone and aponeuroses with the knife. Protargol, while undoubtedly the best agent of to-day for the treatment of fistulous tracts, is no substitute for the knife. In all cases of this sort where main reliance was placed upon protargol only, signal failure resulted; while the combination of the knife, proper drainage, and protargol gave results vastly superior to the simple application of knife and drainage, or injections of bichloride of mercury, liq. villati, argenti nitrat, etc.

It certainly would be unreasonable to expect protargol to replace recognized surgical necessities, known to be the conditio sine qua non in the treatment of fistulæ. Under its influence the purulent infiltration of the tissues and its pain disappear rapidly, sluggish granulations are changed into healthy ones, secretions are diminished, excessive cell proliferation is checked, and thus cicatrization encouraged.

8. *Balanitis.* Eleven cases treated. This trouble is commonly met with in dogs, and was readily healed by 10 per cent. injections three to four times a week. As a rule, but few injections were necessary to reduce the affection to a slight catarrhal state.

9. *Fistula of the spermatic cord.* Seven cases. In all instances protargol injections promptly reduced the phlegmonous state. Twice the fistula closed, to remain so for about two months. Finally amputation of the cord with the écraseur was instituted.

10. *Parenchymatous mastitis.* Thirteen cases, all cows. Beside saline purges, hot fomentations, frequent milking, injections of a 2 per cent. protargol solution was ordered every two hours. Complete recovery on an average in five days. Microscopical examination revealed no pus in the milk at the fifth day of the injection.

11. *Purulent parenchymatous mastitis.* Six months ago the writer was consulted in regard to a dairy herd where all the cows within one week had swollen udders. The affection set in suddenly, the udder hard and very painful, the animals lame more or less, and the skin covering the udder very red. Lactation practically suspended. The milk appears coagulated, containing red blood cells and pus corpuscles. In the older cases the secretion is practically all pus, while coagula obstruct the lacteal ducts. Cessation of appetite and rumination; temperature, 104° to 107° F. The microscopical examination revealed red and white blood cells in great numbers; pus cells and colostrum corpuscles. Based upon this ensemble, the diagnosis of mastitis parenchymatosa apostematosa was made.

Outside of general and local disinfection, isolation of the diseased ones, keeping a separate attendant for these, the main therapeutic agent was a 5 per cent. protargol solution injected every two or three hours into the teats, with subsequent massage. The results were excellent; the pain and swelling subsided, and in almost all cases was gone on the fifth and sixth day. Of course, an additional abscess was lanced. One cow went dry entirely, while another lost the use of one-quarter.

12. *Fistula of the tail.* Animal used to clamp the reins. To do away with this vice, myotomy of the coccygeal depressors was performed; ten days later an abscess formed above the point of incision. This was opened by the surgeon, who performed the myotomy, and the owner informed to pay no attention to it, as the wound would now close. Three weeks after the abscess had been opened the writer saw the animal the first time, receiving the above history. Since the owner seriously objected to any more cutting, he was given a 20 per cent. protargol water-glycerin preparation and told to inject it three times daily. After injecting the material for one week the bottle was broken, the fistula closed, and remained so since (three months).

13. *Dermatitis eczematosa, commonly termed scratches.* The mere fact that almost every horse owner has a "sure cure" for scratches, and at the same time always is on the lookout for something more reliable, is suggestive of the fact that some safe preparation is desirable. The writer has treated any number of this form of dermatitis, with excellent results, by means of a 10 to 20 per cent. protargol-lanolin ointment. After the scurf has been removed by hot fomentations or otherwise, the protargol-lanolin ointment is spread upon some cotton or linen cloth and placed upon the diseased skin and held in place by a bandage, to be removed and renewed once daily. Depending upon the amount of tissue destroyed, four to fourteen days are required to heal this condition. The best results are necessarily obtained when the animal can be kept at rest. The same treatment is equally efficient in gangrenous dermatitis after the necrosed piece has been separated. Protargol in ointment form gave better results than keeping the parts moist with a protargol solution, or dusting them with the pure powder. Only in those cases where excessive granulations threaten, the application of the powder is preferable, as the pressure exerted upon the granulations by the hard crust formed by the protargol greatly helps to form a nice clean scar. Fistulous tracts so often accompanying gangrenous dermatitis heal readily with 10 per cent. protargol injections made three times daily.

14. *Chronic grease, dermatitis chronica verrucosa,* protargol gave only negative results in nine cases.

(To be continued.)

